# Sumoylation of Flotillin-1 promotes EMT in metastatic prostate cancer by suppressing Snail degradation

**DOI:** 10.1038/s41388-018-0641-1

**Published:** 2019-01-10

**Authors:** Donghwan Jang, Hayeong Kwon, Moonjeong Choi, Jaewoong Lee, Yunbae Pak

**Affiliations:** Division of Life Science, Graduate School of Applied Life Science (BK21 Plus Program), PMBBRC, Gyeongsang National University, Jinju, 52828 South Korea

**Keywords:** Sumoylation, Metastasis

## Abstract

Flotillin-1 (Flot-1) has been shown to regulate cancer progression, but the regulatory role of post-translational modifications of Flot-1 on cancers remains elusive. Herein, we show that up-regulated E2 conjugating enzyme UBC9 sumoylates Flot-1 at Lys-51 and Lys-195 with small ubiquitin-like modifier (SUMO)-2/3 modification in metastatic prostate cancer. Mitogen induced the sumoylation and nuclear translocation of Flot-1. The nuclear-targeted Flot-1 physically interacted with Snail, and inhibited Snail degradation through the proteasome in a sumoylation-dependent manner, thereby promoting epithelial-to-mesenchymal transition (EMT). Sumoylation of Flot-1 by up-regulated UBC9 in human metastatic prostate cancer tissues and prostate cancer cells with high metastatic potential positively correlated with the stabilization of Snail and the induction of Snail-mediated EMT genes in the metastatic prostate cancer. Our study reveals a new mechanism of sumoylated Flot-1-mediating Snail stabilization, and identifies a novel sumoylated Flot-1-Snail signaling axis in EMT of metastatic prostate cancer.

## Introduction

Flotillin-1 (Flot-1), a lipid rafts protein, has been shown to regulate cell proliferation [1, 2], and signal transduction by receptor tyrosine kinases including insulin-like growth factor-1 receptor (IGF-1R) [3–10]. Recently, we have shown that palmitoylation of Flot-1 at Cys-34 in the endoplasmic reticulum (ER) is required for the transport of IGF-1R with the Flot-1 to the plasma membrane (PM), and IGF-1-induced Flot-1 palmitoylation turnover in the PM prolongs IGF-1R signaling activation to promote cancer cell proliferation [3].

Epithelial-to-mesenchymal transition (EMT) is a key process for cancer metastasis [11]. Snail is a major transcription factor inducing EMT [12], and its up-regulation in tumor tissues of patients is related to cancer metastasis and recurrence of various cancers [13]. Stability and subcellular localization of Snail, a highly unstable protein, are regulated by GSK-3β-mediated phosphorylation in various cancer cells [14]. Although Snail has been shown to regulate motility and invasive capacity as prostate cancer progresses [15–17], and protein kinase D1 to suppress EMT through phosphorylation of Snail [18], the regulatory mechanisms of stability and subcellular localization of Snail in prostate cancer have remained elusive.

Up-regulation of Flot-1 was reported in prostate and breast cancers, esophageal squamous cells and renal cell carcinomas, and was related to the development and progression of the cancers [2, 19–23]. Santamaría et al. showed that Flot-1 with mitogenic activity induced prostate cancer cell proliferation, which required nuclear translocation, but not palmitoylation of Flot-1 [2]. Nevertheless, the regulatory mechanisms by post-translational modifications, and nuclear translocation of Flot-1 in prostate cancer progression and metastasis remain to be explored.

Here, we show that (i) Lys-51 and Lys-195 of Flot-1 are sumoylated by UBC9 with small ubiquitin-like modifier (SUMO)-2/3 modification, and the sumoylated Flot-1 originating from the non-palmitoylable Flot-1 translocates to the nucleus in mitogenic response; (ii) the nuclear-targeted sumoylated Flot-1 promotes EMT of metastatic prostate cancer by impeding proteasomal degradation of Snail through direct interaction; and (iii) sumoylation of Flot-1 by up-regulated UBC9 in human metastatic prostate cancer tissues and prostate cancer cells with high metastatic potential positively correlates with the stabilization of Snail and the induction of Snail-mediated EMT genes in the metastatic prostate cancer.

## Results

### Flot-1 is sumoylated by UBC9 with SUMO-2/3

**Fig. 1:**
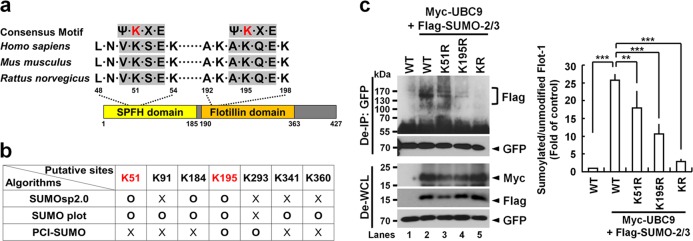
Flot-1 is a sumoylation target protein. **a** Flot-1 contains two SUMO consensus site Lys at 51 and 195. **b** Flot-1 sumoylation sites by prediction algorithms. **c** Immunoblot analysis of denatured immunoprecipitation (De-IP) performed with anti-GFP antibody and whole-cell lysates (De-WCL) from WT Flot-1-GFP-, or sumoylation-defective Flot-1-K51R-GFP-, Flot-1-K195R-GFP- or Flot-1-K51R/K195R-GFP (KR)-expressing PC3 cells transfected with or without Myc-UBC9 and Flag-SUMO-2/3. The densitometry ratios of sumoylated Flot-1 to unmodified Flot-1 are illustrated (mean ± s.d., *n* = 3, **P < 0.01, ***P < 0.001, two-way ANOVA)

The majority of the sumoylation sites are within a consensus motif, ψ-K-X-E/D (ψ is a hydrophobic amino acid) [24]. Flot-1 has two potential sumoylation sites, Lys-51 within SPFH domain and Lys-195 within Flotillin domain (Fig. 1a). Three sumoylation prediction algorithms, SUMOsp2.0 [25], SUMO plot (http://www.abgent.com/sumoplot), and PCl-SUMO [26] predicted Lys-51 and Lys-195 to be potential sumoylation sites (Fig. 1b). Accordingly, sumoylation-defective K51R, K195R, and K51/195R mutants were generated and tested. Denatured immunoprecipitation (De-IP) analysis showed that 70-kDa wild-type (WT) Flot-1-GFP is sumoylated to 130–170-kDa bands upon ectopic transfection of Myc-tagged E2 conjugating enzyme UBC9 and Flag-tagged SUMO-2/3 (Fig. 1c, lanes 1 vs. 2) in WT Flot-1-GFP-expressing PC3 cells. Flot-1 sumoylation in Flot-1-K51R-GFP- or Flot-1-K195R-GFP-expressing cells was reduced by 7.8-fold and 15.1-fold, respectively, and almost no Flot-1 sumoylation was detected in Flot-1-K51R/K195R-GFP (KR)-expressing cells as compared to that in WT Flot-1-expressing cells (Fig. 1c, lanes 2 vs. 3, 4, and 5). The results show that both Lys-51 and Lys-195 residues are SUMO-2/3 modification sites for UBC9-mediated sumoylation of Flot-1 in PC3 cells.

### Mitogen-response Flot-1 sumoylation and UBC9 up-regulation correlate with Snail up-regulation in prostate cancer cells with high metastatic potential

**Fig. 2:**
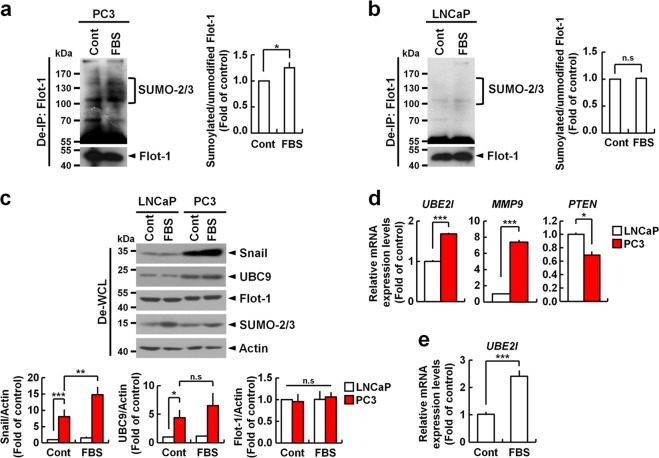
Mitogen-response Flot-1 sumoylation and *UBE2I* overexpression correlate with Snail up-regulation in prostate cancer cells with high metastatic potential. **a–c** Endogenous Flot-1 sumoylation by UBC9 with SUMO-2/3. Immunoblot analysis of De-IP performed with anti-Flot-1 antibody and De-WCL from PC3 and LNCaP cells stimulated with (FBS) or without (Cont) 10% FBS for 6 h. The densitometry ratios of **a**, **b** sumoylated Flot-1 to unmodified Flot-1, and **c** Snail, UBC9, and Flot-1 to actin are illustrated (n.s. not significant; mean ± s.d., *n* = 3, *P < 0.05, **P < 0.01, ***P < 0.001, Student’s *t* test (**a**, **b**) and two-way ANOVA (**c**)). **d**, **e** qRT-PCR analysis of **d** LNCaP and PC3 cells for *UBE2I*, *MMP9*, and *PTEN* expression, and **e** PC3 cells stimulated with or without 10% FBS for 6 h for *UBE2I* expression. Data are represented as mean ± s.d. (*n* = 3, *P < 0.05, ***P < 0.001, Student’s *t* test)

To explore the regulatory role of Flot-1 sumoylation in prostate cancer, we examined mitogen-response sumoylation of Flot-1, and its relation to the metastatic potential, comparing PC3 and LNCaP cells with high and low metastatic potential [27], respectively. When serum-deprived quiescent cells were stimulated with serum, sumoylation (110–150-kDa) of endogenous Flot-1 (48-kDa) was increased by 1.3-fold in PC3, but not in LNCaP cells (Fig. 2a, b). Of note, basal expression levels of Snail and E2 conjugating enzyme UBC9 were markedly elevated by 8.1-fold and 4.4-fold, respectively in PC3 cells as compared to LNCaP cells, and the Snail level was increased by 1.8-fold upon serum stimulation in PC3, but not in LNCaP cells (Fig. 2c and Supplementary Fig. S1a). There were no significant difference and change in Flot-1 expression level between PC3 and LNCaP cells and upon serum stimulation (Fig. 2c and Supplementary Fig. S1a). In PC3, compared to LNCaP cells, *UBE2I* (UBC9) and *MMP9* (MMP9) were up-regulated by 1.7-fold and 7.4-fold, respectively, and *PTEN* (PTEN) was down-regulated (Fig. 2d). Serum stimulation further increased the *UBE2I* induction by 2.4-fold in PC3 cells (Fig. 2e). DU145 cells, another prostate cancer cells with high metastatic potential [27], also exhibited the elevated expression of Snail and UBC9 (Supplementary Fig. S1a), and the mitogen-response sumoylation of Flot-1 by SUMO-2/3 (Supplementary Fig. S1a, b). These results show that mitogen-response Flot-1 sumoylation along with UBC9 up-regulation correlates with the up-regulation of Snail and induction of EMT-related genes, which are regulated by Snail, in prostate cancer cells with high metastatic potential.

### Sumoylation is required for mitogen-stimulated translocation of Flot-1 to the nucleus

**Fig. 3:**
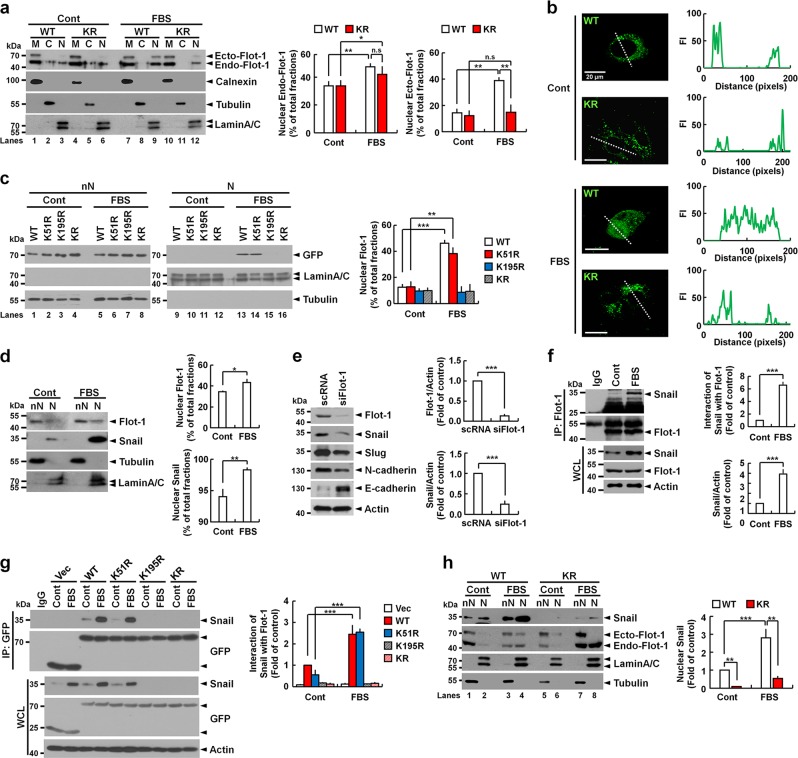
Sumoylated Flot-1 translocates to the nucleus and up-regulates Snail in response to mitogen. **a** Immunoblot analysis of membrane (M), cytosol (C), and nuclear (N) fractions and **b** confocal microscopy analysis of WT Flot-1-GFP- or Flot-1-KR-GFP-expressing PC3 cells stimulated with or without 10% FBS for 6 h. **a** The amounts of endogenous Flot-1 and ectopic WT Flot-1-GFP or Flot-1-KR-GFP present in nuclear fraction are presented with 100% representing the total (M + C + N) fractions (n.s. not significant; mean ± s.d., *n* = 3, *P < 0.05, **P < 0.01, two-way ANOVA). Ecto ectopic, Endo endogenous. **b** Representative single optical sections are shown. The dotted white line in each panel was converted to fluorescence intensity (FI) of line profiles. **c**, **d**, **h** Immunoblot analysis of non-nuclear (nN) and nuclear (N) fractions from **d** PC3 cells and **c**, **h** WT Flot-1-GFP-, Flot-1-K51R-GFP-, Flot-1-K195R-GFP-, or Flot-1-KR-GFP-expressing PC3 cells stimulated with or without 10% FBS for 6 h. The amounts of ectopic WT Flot-1-GFP, Flot-1-K51R-GFP, Flot-1-K195R-GFP, or Flot-1-KR-GFP, and endogenous Flot-1 and Snail present in nuclear fraction are presented with 100% representing the total (nN + N) fractions (mean ± s.d., *n* = 3, *P < 0.05, **P < 0.01, ***P < 0.001, two-way ANOVA (**c**, **h**) and Student’s *t* test (**d**)). **e** Knock-down of endogenous Flot-1 attenuates the expression of EMT-related markers. Immunoblot analysis of WCL from scRNA- or siFlot-1-expressing PC3 cells. The densitometry ratios of Flot-1 and Snail to actin are illustrated (mean ± s.d., *n* = 3, ***P < 0.001, Student’s *t* test). **f** Serum-induced endogenous interaction between Flot-1 and Snail. Immunoblot analysis of IP performed with anti-Flot-1 antibody and WCL from PC3 cells stimulated with or without 10% FBS for 6 h. The densitometry ratios of Flot-1 interaction with Snail, and Snail to actin are illustrated (mean ± s.d., *n* = 3, ***P < 0.001, Student’s *t* test). **g** Sumoylation at Lys-195 is necessary for interaction with Snail. Immunoblot analysis of IP performed with anti-GFP antibody and WCL from GFP-vector (Vec)-, WT Flot-1-GFP-, Flot-1-K51R-GFP-, Flot-1-K195R-GFP-, or Flot-1-KR-GFP-expressing PC3 cells stimulated with or without 10% FBS for 6 h. The densitometry ratios of Flot-1 interaction with Snail are illustrated (mean ± s.d., *n* = 3, ***P < 0.001, two-way ANOVA)

In response to serum stimulation, nuclear-translocated Flot-1 promoted PC3 cell proliferation with mitogenic activity [2]. We tested if sumoylation is required for serum-stimulated nuclear targeting of Flot-1 in PC3 cells. In serum-deprived quiescent cells, endogenous and ectopic WT Flot-1 and sumoylation-defective KR mutant were predominantly detected in the membrane fraction (Fig. 3a, lanes 1 and 4). Upon serum stimulation, endogenous and ectopic WT Flot-1, but not KR mutant, were detected in nuclear fraction (Fig. 3a, lanes 9 and 12). Confocal microscopy analysis validated that WT Flot-1, but not KR mutant, translocates to the nucleus after serum stimulation (Fig. 3b). Further investigation showed that WT Flot-1 and K51R mutant were translocated in nuclear fraction after serum stimulation, but K195R and KR mutants were not (Fig. 3c, lanes 13 and 14 vs. 15 and 16). These results show that sumoylation of Lys-195 is important for mitogen-stimulated translocation of Flot-1 to the nucleus in PC3 cells.

### Sumoylation is required for Flot-1 to interact with and up-regulate Snail

Serum stimulation increased nuclear localization of endogenous Flot-1, and markedly elevated nuclear Snail level in PC3 cells (Fig. 3d). Knock-down of endogenous Flot-1 reduced levels of Snail by 4.0-fold and Slug. N-cadherin was decreased but E-cadherin was significantly elevated as compared to control (Fig. 3e). We thus scrutinized how the nuclear-targeted sumoylated Flot-1 controls the elevation of Snail level in the nucleus. Serum stimulation induced direct interaction of Flot-1 with up-regulated Snail endogenously (Fig. 3f), and the interaction required Lys-195 sumoylation of Flot-1 (Fig. 3g). In WT Flot-1-expressing cells, Snail level in the nuclear fraction was increased by 2.8-fold after serum stimulation (Fig. 3h, lanes 2 vs. 4). But there was no such increase of nuclear Snail level in KR mutant-expressing cells (Fig. 3h, lanes 4 vs. 8). The specific requirement of Lys-195 sumoylation of Flot-1 for the Snail up-regulation in the nucleus was further validated in Supplementary Fig. S2. These results show that mitogen-induced nuclear translocation and interaction with Snail of sumoylated Flot-1 are positively correlated in prostate cancer cells with high metastatic potential.

### Sumoylation of non-palmitoylable Flot-1 is responsible for interaction with and stabilization of Snail

**Fig. 4:**
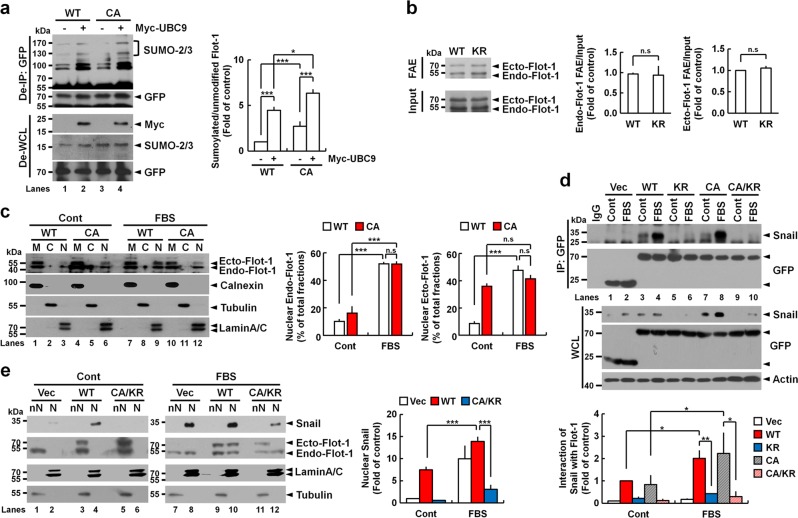
Sumoylation of non-palmitoylable Flot-1 coordinates mitogen-response nuclear translocation and interaction with Snail in the nucleus. **a** Non-palmitoylable Flot-1 is more susceptible to sumoylation. Immunoblot analysis of De-IP performed with anti-Flot-1 antibody and De-WCL from WT Flot-1-GFP- or palmitoylation-defective Flot-1-CA-GFP-expressing PC3 cells transfected with or without Myc-UBC9. The densitometry ratios of sumoylated Flot-1 to unmodified Flot-1 are illustrated (mean ± s.d., *n* = 3, *P < 0.05, ***P < 0.001, two-way ANOVA). **b** FAE analysis of WT Flot-1-GFP- or Flot-1-KR-GFP-expressing PC3 cells shows that sumoylation status does not affect palmitoylation of Flot-1. The densitometry ratios of FAE to Input are illustrated (n.s. not significant; mean ± s.d., *n* = 3, Student’s *t* test). Ecto ectopic, Endo endogenous. **c** Nuclear localization of non-palmitoylable Flot-1, independently of serum stimulation. Immunoblot analysis of M, C, and N fractions from WT Flot-1-Flag- or Flot-1-CA-Flag-expressing PC3 cells stimulated with or without 10% FBS for 6 h. The amounts of endogenous Flot-1 and ectopic WT Flot-1-Flag or Flot-1-CA-Flag present in nuclear fraction are presented with 100% representing the total (M + C + N) fractions (n.s. not significant; mean ± s.d., *n* = 3, ***P < 0.001, two-way ANOVA). Ecto ectopic, Endo endogenous. **d**, **e** Non-palmitoylable but sumoylated Flot-1 interacts with and up-regulates nuclear Snail. Immunoblot analysis of **d** IP performed with anti-GFP antibody and WCL from GFP-vector (Vec)-, WT Flot-1-GFP-, Flot-1-KR-GFP-, Flot-1-CA-GFP-, or Flot-1-CA/KR-GFP-expressing PC3 cells, and **e** nN and N fractions from GFP-vector-, WT Flot-1-GFP-, or Flot-1-CA/KR-GFP-expressing PC3 cells stimulated with or without 10% FBS for 6 h. **d** The densitometry ratios of Flot-1 interaction with Snail are illustrated (mean ± s.d., *n* = 3, *P < 0.05, **P < 0.01, two-way ANOVA). **e** The amount of Snail present in nuclear fraction is presented with 100% representing the total (nN + N) fractions (mean ± s.d., *n* = 3, ***P < 0.001, two-way ANOVA). Ecto ectopic, Endo endogenous

Palmitoylation at Cys-34 is important for intracellular localization of Flot-1 [3, 28, 29]. We have previously shown that palmitoylation-defective Flot-1-C34A (CA) mutant is unable to exit and accumulates in the ER [3]. We tested whether the status of Cys-34 palmitoylation influences Flot-1 sumoylation in PC3 cells. Sumoylation rate of CA mutant by endogenous UBC9 and SUMO-2/3 was 2.7-fold higher than that of WT Flot-1 (Fig. 4a, lanes 1 vs. 3), and was further increased upon ectopic overexpression of UBC9 (Fig. 4a, lanes 3 vs. 4). On the contrary, sumoylation did not affect Flot-1 palmitoylation, as demonstrated by fatty acyl biotin exchange (FAE) assay that both WT and KR mutant were equally palmitoylated (Fig. 4b). These results suggest that sumoylation of Flot-1 depends on palmitoylation state of Flot-1; non-palmitoylable Flot-1 is much more prone to the sumoylation by UBC9 and SUMO-2/3.

Palmitoylation at Cys-34 was not required for serum-stimulated Flot-1 translocation to the nucleus in PC3 cells [2]. In agreement with the report, nuclear localization of ectopic CA mutant was observed. However, in contrast that endogenous Flot-1 was translocated to the nucleus upon serum stimulation, CA mutant did not exhibit serum stimulation-dependent nuclear localization (Fig. 4c, lanes 6 vs. 12). Serum stimulation increased WT and CA mutant interaction with Snail and Snail expression in WT Flot-1 and CA mutant-expressing cells (Fig. 4d, lanes 3, 4, 7, and 8). But KR and CA/KR mutants did not interact with Snail with or without serum stimulation, and there was no increased Snail expression detected upon serum stimulation in KR or CA/KR mutant-expressing cells (Fig. 4d, lanes 5, 6, 9, and 10). Serum-stimulated nuclear localization of Flot-1 and up-regulation of nuclear Snail were observed in vector- and WT Flot-1-expressing cells, but were significantly reduced in CA/KR mutant-expressing cells (Fig. 4e, lanes 8 and 10 vs. 12). These results show that sumoylation of Flot-1 originating from the non-palmitoylable Flot-1 and nuclear translocation of the sumoylated Flot-1 in response to mitogenic stimulation positively regulate Snail stability in the nucleus through direct interaction in prostate cancer cells with high metastatic potential.

### Sumoylated Flot-1 stabilizes Snail by suppressing proteasomal degradation

**Fig. 5:**
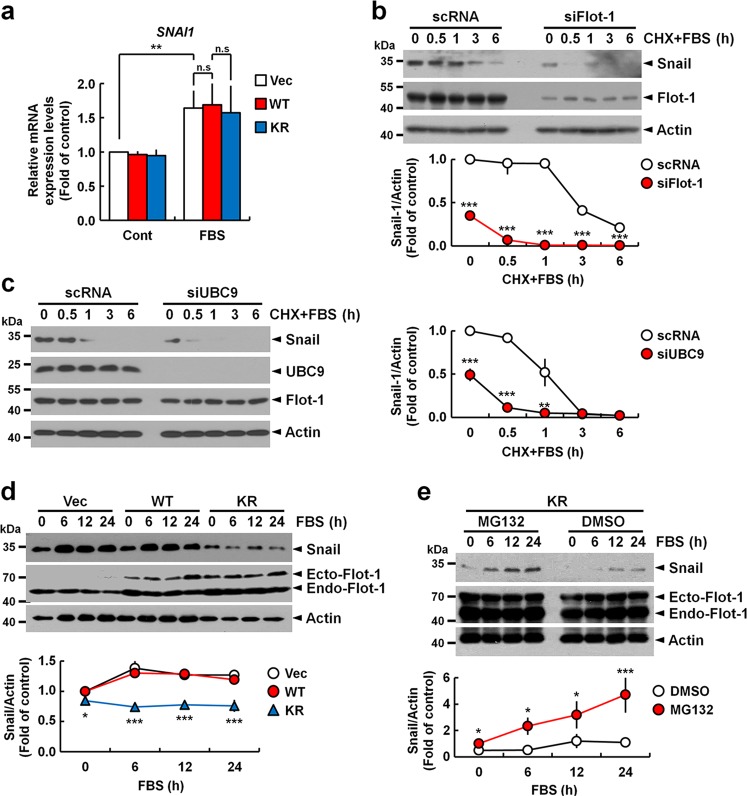
Sumoylation of Flot-1 promotes Snail stability by inhibiting proteasomal degradation. **a** Sumoylation of Flot-1 does not affect serum-stimulated *SNAI1* induction. qRT-PCR analysis of GFP-vector (Vec)-, WT Flot-1-GFP-, or Flot-1-KR-GFP-expressing PC3 cells stimulated with or without 10% FBS for 6 h for *SNAI1* expression. Data are represented as mean ± s.d. (n.s. not significant; *n* = 3, **P < 0.01, two-way ANOVA). **b**, **c** Depletion of endogenous Flot-1 and UBC9 causes rapid degradation of Snail. Immunoblot analysis of WCL from scRNA-, siFlot-1-, or siUBC9-expressing PC3 cells stimulated with 10% FBS in the presence of 50 μM CHX for indicated times. The densitometry ratios of Snail to actin are illustrated (mean ± s.d., *n* = 3, **P < 0.01, ***P < 0.001, two-way ANOVA). **d**, **e** Sumoylated Flot-1 suppresses proteasomal degradation of Snail. Immunoblot analysis of WCL from **d** GFP-vector-, WT Flot-1-GFP-, or Flot-1-KR-GFP-expressing PC3 cells stimulated with 10% FBS for indicated times, and from **e** Flot-1-KR-GFP-expressing cells treated with 10 μM MG132 or DMSO for 4 h and stimulated with 10% FBS for indicated times. The densitometry ratios of Snail to actin are illustrated (mean ± s.d., *n* = 3, *P < 0.05, ***P < 0.001, two-way ANOVA). Ecto ectopic, Endo endogenous

Serum stimulation induced almost the same level of *SNAI1* transcript in vector-, WT Flot-1-, and KR mutant-expressing PC3 cells (Fig. 5a), which indicated that sumoylation of Flot-1 contributes to the sustenance of Snail protein stability. To explore dynamics of Snail stability control by sumoylated Flot-1, we investigated the kinetics of Snail degradation after serum stimulation in the presence of cycloheximide (CHX), an inhibitor of protein biosynthesis, and tested the effects of depletion of endogenous Flot-1 (Fig. 5b) and UBC9 (Fig. 5c) on the kinetics. Snail was stable until 0.5–1 h serum stimulation, and degradation of Snail started thereafter in control. However, Snail in Flot-1- or UBC9-depleted cells underwent rapid and complete degradation within 30 min of serum stimulation (Fig. 5b, c). The elevated Snail level was stably maintained during 6–24 h after serum stimulation in vector control- and WT Flot-1-expressing cells. But KR mutant-expressing cells exhibited attenuated Snail stability over the time-course (Fig. 5d). However, treatment with MG132, a proteasomal protein degradation inhibitor, restored serum-stimulated elevation and stable maintenance of the elevated Snail expression in KR mutant-expressing cells (Fig. 5e). These results show that nuclear-targeted sumoylated Flot-1 prevents rapid proteasomal degradation of Snail, and hence promotes Snail stability in prostate cancer cells with high metastatic potential.

### UBC9-mediated sumoylation of Flot-1 promotes Snail-induced EMT in metastatic prostate cancer

**Fig. 6:**
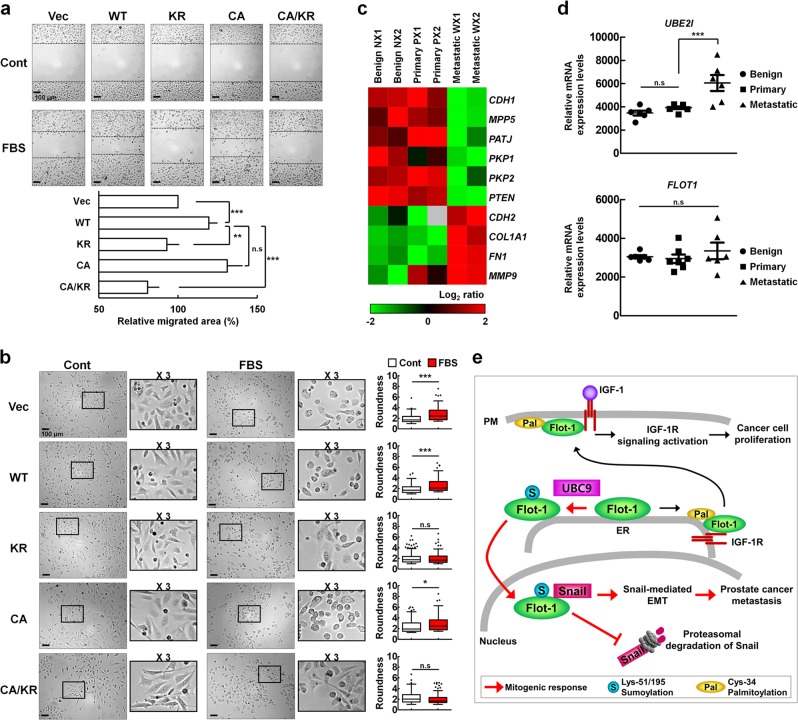
Sumoylation of Flot-1 by up-regulated *UBE2I* promotes Snail-mediated EMT in metastatic prostate cancer. **a**, **b** Non-palmitoylable but sumoylated Flot-1 promotes serum-stimulated migration of and EMT-like morphology in prostate cancer cells. **a** Migration assay and **b** phase-contrast microscopy analysis of GFP-vector (Vec)-, WT Flot-1-GFP-, Flot-1-KR-GFP-, Flot-1-CA-GFP-, or Flot-1-CA/KR-GFP-expressing PC3 cells stimulated with or without 10% FBS for 24 h. **a** The area of migrated cells in response to serum stimulation based on the obtained images was presented with 100% representing the vector control (n.s. not significant; mean ± s.d., *n* = 9, **P < 0.01, ***P < 0.001, two-way ANOVA). **b** Representative single optical sections are shown. Magnifications (×3) of the areas framed in the images are shown. EMT morphological changes are quantified by calculating the roundness parameter (Perimeter^2^/(4 × *π* × area)) of each cell from FBS-untreated control group (white square) and FBS-treated group (red square) (n.s. not significant; mean ± s.d., *n* = 3, *P < 0.05, ***P < 0.001, Student’s *t* test). **c** Expression of EMT-related genes induced by Snail is positively correlated in human metastatic prostate cancer tissues. Six pooled samples from benign (NX1, NX2), primary (PX1, PX2), or metastatic (WX1, WX2) prostate cancer tissues were analyzed by the heat map of log_2_ gene expression of the EMT-related genes induced by Snail. Red indicates high and green low levels of transcript abundance. **d** Up-regulation of *UBE2I* in human metastatic prostate cancer tissues. Total 19 individual benign (*n* = 6), primary (*n* = 7), and metastatic (*n* = 6) prostate cancer samples were analyzed for *FLOT1* and *UBE2I* expression (n.s. not significant; mean ± s.d., ***P < 0.001, one-way ANOVA). **e** A model for the regulation of Snail-mediated EMT by Flot-1 sumoylation in metastatic prostate cancer. Upon mitogen stimulation, non-palmitoylable Flot-1 is sumoylated at Lys-51 and Lys-195 by UBC9 and SUMO-2/3 in the ER, and translocates to the nucleus. The nuclear-targeted sumoylated Flot-1 physically binds with Snail to block proteasomal degradation of Snail, and thereby boosts the metastatic potential of prostate cancer through the Snail-mediated EMT process. Cys-34-palmitoylated Flot-1 in the ER translocates together with IGF-1R to the PM. The PM-targeted Cys-34-palmitoylated Flot-1 sustains IGF-1-induced IGF-1R signaling activation to prolong cancer cell proliferation [3]

Serum stimulation increased PC3 cell migration by 19.4 ± 5.6% and 31.1 ± 9.7% in WT Flot-1- and CA mutant-expressing cells, respectively, as compared to control vector-expressing cells. But, both KR mutant- and CA/KR mutant-expressing cells exhibited retardation of serum-stimulated cell migration (Fig. 6a). In accordance with the results, control vector-, WT Flot-1- and CA mutant-expressing cells displayed typical epithelial morphology after serum deprivation, but underwent changes to the mesenchymal-like morphology after serum stimulation (Fig. 6b). KR mutant- and CA/KR mutant-expressing cells, however, displayed both clustered epithelial-like cells and spindle-shaped mesenchymal-like cells after serum stimulation (Fig. 6b), indicating retarded serum-induced EMT. Quantitative morphological assessment of EMT evaluated by calculating the roundness of each cell in images revealed no significant changes in the KR mutant- and CA/KR mutant-expressing cells after serum stimulation (Fig. 6b).

Expression profile analysis of EMT-related genes in human benign prostate and primary and metastatic prostate cancer tissues, using the mRNA microarray data [30] downloaded from the Gene Expression Omnibus (GEO) database (accession number: GSE3325), showed that the expression of *CDH1* (E-cadherin), *MPP5* (PALS1), *PATJ* (PATJ), *PKP1* (desmoplakin), *PKP2* (plakophilin), and *PTEN* (PTEN), the genes known to be down-regulated by Snail [31, 32] were suppressed, while *CDH2* (N-cadherin), *COL1A1* (collagen), *FN1* (fibronectin), and *MMP9* (MMP9), the genes known to be up-regulated by Snail [31] were highly expressed in metastatic prostate cancer tissues (Fig. 6c). *UBE2I* (UBC9) expression was significantly higher in metastatic prostate cancer tissues than in benign and primary prostate cancer tissues (Fig. 6d). Thus, our analysis showed that Snail-mediated EMT inducing genes and *UBE2I* are coordinately overexpressed in metastatic prostate cancer. Consistent with the results shown in Fig. 2c and Supplementary Fig. S1a, there was no significant difference of *FLOT1* (Flot-1) expression among benign and primary and metastatic prostate cancer tissues (Fig. 6d). Together, these results show that sumoylation of Flot-1 by up-regulated UBC9 in metastatic prostate cancer tissues and prostate cancer cells with high metastatic potential positively correlates with the stabilization of Snail and the induction of Snail-mediated EMT genes in the metastatic prostate cancer.

## Discussion

Post-translational modifications provide a key functional regulatory link to tumorigenesis and cancer progression and metastasis. Intracellular signaling proteins such as p53, STAT1, and IκBα have been shown to be modified by sumoylation, thereby contributing to carcinogenesis [33]. However, the regulatory mechanisms of sumoylation, and the interplay of sumoylated proteins with intracellular signaling proteins in cancer progression and metastasis remain unclear.

The present study identifies that Flot-1 is a novel sumoylation target protein, and sumoylation of Flot-1 positively regulates Snail stability to promote EMT of metastatic prostate cancer. Figure 6e illustrates a proposed model for the control of Snail-mediated EMT by Flot-1 sumoylation. In response to mitogen, Flot-1 is sumoylated at Lys-51 and Lys-195 by UBC9 with SUMO-2/3 modification in the ER, and the sumoylated Flot-1 is translocated to the nucleus. Nuclear-targeted sumoylated Flot-1 associates with Snail to block proteasomal degradation of Snail, thereby boosting the metastatic potential of prostate cancer via Snail-mediated EMT.

Santamaría et al. showed that prostate tumor overexpressed 1 (PTOV1) assisted nuclear translocation of Flot-1, and both proteins were required for PC3 cell proliferation, although Flot-1 depletion did not affect nuclear localization of PTOV1 [2]. Our investigation on if mitogen-response sumoylation of Flot-1 is crucial for interaction with PTOV1 showed that their interaction requires Lys-195 sumoylation of Flot-1, irrespective of mitogen stimulation (Supplementary Fig. S3). Given that Lys-195 sumoylation of Flot-1 is necessary for mitogen-response nuclear translocation and interaction with Snail (Fig. 3c, g), our data suggest that Lys-195 sumoylated Flot-1 stabilizes nuclear Snail in cooperation with PTOV1. Of interest, PC3 cell proliferation was unaffected in non-sumoylable KR mutant-expressing cells in response to IGF-1 and serum stimulation, but was attenuated in non-palmitoylable CA mutant-expressing cells (Supplementary Fig. S4a). And there was no indication of any alteration on PTOV1 expression level in sumoylation-defective mutant-expressing PC3 cells (Supplementary Fig. S3). Collectively, the report by Santamaría et al. [2] and our results indicate that PTOV1 independently of Flot-1 could possibly regulate PC3 cell proliferation.

It has been shown that Flot-1 associates with lipid rafts in the PM, and palmitoylation on Cys-34 directs PM targeting and protein interactions [3, 28, 29]. We have recently shown that the palmitoylation of Flot-1 is indispensable for the ER exit and the PM localization of IGF-1R together with the Flot-1 [3] (Fig. 6e). In that report, we also showed that IGF-1 induces depalmitoylation and repalmitoylation, a dynamic palmitoylation turnover, of the PM-targeted Flot-1, which facilitates prolonged activation of IGF-1R on the cell surface and hence promotes cervical cancer cell proliferation [3]. As shown in Supplementary Fig. S4a, IGF-1 induced PC3 cell proliferation, and the proliferation was increased by WT Flot-1 or KR mutant, but not CA mutant overexpression, suggesting that palmitoylation, but not sumoylation, of Flot-1 is responsible for IGF-1R signaling-mediated prostate cancer cell proliferation. However, IGF-1-induced PC3 cell migration was increased by WT Flot-1, but not KR mutant overexpression (Supplementary Fig. S4b). The present study shows that the sumoylated Flot-1 originating from the non-palmitoylable Flot-1 translocates to the nucleus in mitogenic response, and is responsible for the Snail stability to enhance metastatic potential in prostate cancer. Sumoylation of Flot-1 depended on palmitoylation state of Flot-1; non-palmitoylable Flot-1 was much more susceptible to sumoylation by UBC9 and SUMO-2/3. Sumoylation status, however, did not affect Flot-1 palmitoylation. We have shown that palmitoylation-defective Flot-1 accumulates in the ER because it is incapable of exiting the ER [3]. The present study shows that palmitoylation is dispensable, but sumoylation is required for mitogen-induced nuclear targeting and interaction with Snail of Flot-1 to function as a positive regulator in Snail-mediated EMT in metastatic prostate cancer. Thus our data indicate that sumoylation of the non-palmitoylable Flot-1 is likely to take place in the ER by ER-localized UBC9, and the sumoylated Flot-1 is translocated to the nucleus to stabilize Snail for the EMT of metastatic prostate cancer in mitogenic response (Fig. 6e). Together, these data suggest that palmitoylation of Flot-1 regulates prostate cancer cell proliferation via IGF-1R signaling activation in the PM, while sumoylation of Flot-1 promotes EMT in metastatic prostate cancer via regulation of Snail stability in the nucleus. Our findings thus provide the underlying molecular mechanisms of IGF-1-induced prostate cancer proliferation, progression, and metastasis controlled by post-translational modifications of Flot-1.

Cell–cell adhesion was promoted by cadherins at cell–cell junction, and perturbation of cell–cell junction was associated with cancer cell invasion and metastasis [34]. Flot microdomains in the PM were required for cadherin stabilization at cell–cell junction [35]. Previous studies showed that Flot localized to cell–cell contact sites [28, 29, 36, 37] and regulated N- and E-cadherin-mediated cell–cell adhesion in mesenchymal and epithelial cells [35]. We observed that depletion of endogenous Flot-1 down-regulated N-cadherin and up-regulated E-cadherin in PC3 cells (Fig. 3e). The expression of *CDH1* (E-cadherin) was suppressed, while *CDH2* (N-cadherin) was highly expressed in metastatic prostate cancer tissues (Fig. 6c). However, no significant difference of *FLOT1* (Flot-1) expression was detected among benign and primary and metastatic prostate cancer tissues, whereas *UBE2I* (UBC9) expression was significantly higher in metastatic prostate cancer tissues than in benign and primary prostate cancer tissues (Fig. 6d). Based on our findings that sumoylated Flot-1 translocates to the nucleus in mitogenic response, and stabilizes Snail for induction of EMT-related genes in metastatic prostate cancer, we would speculate that palmitoylated Flot-1 localized in the PM microdomains might regulate cadherin stabilization at cell–cell junction, presumably via a dynamic palmitoylation turnover of the PM-targeted Flot-1. However, the possible role of post-translational modifications of Flot-1 in cadherin-mediated cell–cell adhesion associated with cancer cell invasion and metastasis remains to be demonstrated.

Our findings provide novel insights into the regulatory cross-talk, and spatio-temporal dynamics between sumoylation and palmitoylation of Flot-1 in prostate cancer cell proliferation, progression, and metastasis. In addition, our findings of the direct correlation between Flot-1 sumoylation and abnormal up-regulation of Snail provide potential implications that Flot-1 sumoylation may serve as a marker for diagnosis of malignant prostate cancer, and a novel therapeutic strategy to control EMT in metastatic prostate cancer by targeting the E2 conjugating enzyme UBC9.

## Materials and methods

### Cell culture

Human prostate adenocarcinoma PC3, derived from bone metastasis, and LNCaP, derived from lymph node metastasis, cells were purchased from the Korean Cell Line Bank. Cells were cultured in RPMI-1640 medium (Thermo Fisher Scientific) containing 10 mM D-glucose supplemented with 10% FBS (ATCC) and 1% penicillin/streptomycin in a 5% CO_2_ incubator at 37 °C. For mitogen stimulation, cells were serum-deprived for 24 h and treated with or without 10% FBS for 6 h. All cells were routinely tested for negative mycoplasma contamination using Mycoplasma Detection Kit (Lonza).

### Plasmids

A full-length *Homo sapiens* Flot-1 cDNA (GeneBank accession number NM_005803.2) was subcloned into pDS-X-Flag and pEGFP-N1 vector as previously described [3]. C34A, K51R, K195R, K51/195R, and C34A/K51/195R mutants were generated by using WT Flot-1-Flag or Flot-1-GFP as a template via EZchange site-directed mutagenesis kit (Enzynomics). All expression vectors were verified by sequencing. Flag-SUMO-2/3 and Myc-UBC9 plasmids were kindly provided by Prof. Chin Ha Chung (Seoul National University). All plasmids were transfected by FuGene^®^ HD (Promega).

### Sumoylation assay

Sumoylation assay with De-IP was performed as described [38]. Denatured whole cell lysates (De-WCL), after boiling for 5 min and diluting to yield 0.1% SDS final concentration using TNESV buffer, were immunoprecipitated with primary antibody for overnight and with Protein G Plus-Agarose beads (Merck Millipore) further for 4 h at 4 °C. The immunoprecipitates were washed in TNESV buffer, resuspended in 2× SDS-PAGE sample buffer, and analyzed by immunoblotting.

### Real-time quantitative RT (qRT)-PCR

Total RNA was extracted with TRIzol reagent (SolGent) and cDNA was generated using Accupower RT PreMix kit (Bioneer). The cDNA was used as the template for the subsequent qRT-PCR analysis using EvaGreen^®^ Supermix (Bio-Rad) and the Eco real-time PCR system (Illumina). qRT-PCR analysis of cDNA sample was normalized to *GAPDH*. The sequences of qRT-PCR primers are listed in Supplementary Table 1.

### Nuclear fractionation

Nuclear fractionation was performed as described [39]. Cells were scraped with hypotonic lysis buffer, homogenized, and centrifuged (1,000*g*, 3 min, 4 °C). The crude nuclear pellet was resuspended in nuclear isolation buffer and incubated on ice for 5 min. The nuclei were pelleted by centrifugation (90*g*, 3 min, 4 °C) to yield the nuclear fraction. The initial supernatant was centrifuged (12,500*g*, 15 min, 4 °C) to yield the crude cytoplasmic fraction and membrane pellet. The crude cytoplasmic fraction was centrifuged (100,000*g*, 60 min, 4 °C) using a TLA 100.3 rotor (Beckman) to yield the pure cytoplasmic fraction. The membrane, cytosol, and nuclear fractions or nuclear and non-nuclear fractions were analyzed by immunoblotting. Calnexin, tubulin, and lamin A/C are used as markers for membrane, cytosol, and nuclear fractions, respectively.

### Confocal microscopy

Cells were fixed with 3.7% paraformaldehyde in PBS for 20 min and rinsed with PBS. Fluorescent images were obtained using an Olympus Fluoview 1000 confocal microscope attached to IX-81 inverted microscope equipped with PlanApo 100×/1.35 oil immersion objective lens (Olympus). GFP signals were excited using an Argon laser at 488 nm. The dotted white lines drawn in the panels were quantificated to plot profiles using the Line Profile Tool of ImageJ (NIH).

### FAE analysis

FAE was performed as described [40, 41]. Cells were lysed with 1% SDS buffer and free cysteines alkylated by overnight incubation at 4 °C with 25 mg/ml N-ethylmaleimide. Protein pellets were resolubilized in 1% SDS buffer, treated with 200 mM hydroxylamine, and incubated with 1 mM Biotin-HPDP for 1 h. Protein pellets were resolubilized in 1% Triton X-100 and 0.2% SDS buffer and incubated with NeutrAvidin-Agarose beads overnight at 4 °C. The beads were collected by centrifugation (13,500*g*, 10 min, 4 °C), followed by four washes in 1% Triton X-100 and 0.2% SDS buffer. Biotinylated proteins were eluted in 5× SDS-PAGE sample buffer and subjected to immunoblotting.

### Immunoprecipitation and immunoblotting

Immunoprecipitation, immunoblotting, and densitometry were performed as previously described [42, 43]. Primary and secondary antibodies used are listed in Supplementary Table S2.

### Knock-down of Flot-1 and UBC9

siRNA target of Flot-1 (siFlot-1) (M-010636-00-0005), UBC9 (siUBC9) (M-004910-00-0005), and scramble control (scRNA) (D-001136-01-05) were purchased from Dharmacon. Transfection of siRNA duplexes was carried out using DharmaFECT3 Transfection Reagent (Dharmacon).

### Migration assay

Cells were seeded into the ibid^®^ cell culture insert each chamber at a density of 1 × 10^4^ cells/chamber on a 24-well plate and allowed to attach for 12 h. After removing the ibid^®^ cell culture insert, the cells were allowed to migrate for 24 h, and images were acquired at an Optika XDS-3FL microscope using the 10× objective. Quantification of migrated cells area based on the obtained images was analyzed using ImageJ (NIH).

### Quantitative morphological assessment of EMT

EMT morphological changes were quantitatively evaluated by calculating the roundness of each cell in images processed by Adobe Photoshop software as described previously [44]. Three images per group were calculated with the morphological parameter (roundness = Perimeter^2^/(4 × *π* × area)) by Image Pro 10.0 software (Media Cybernetics). The total cell numbers per group assessed were as follows: GFP-vector-expressing group (FBS-untreated cell numbers = 356, FBS-treated cell numbers = 375); WT Flot-1-GFP-expressing group (FBS-untreated cell numbers = 441, FBS-treated cell numbers = 406); Flot-1-KR-GFP-expressing group (FBS-untreated cell numbers = 476, FBS-treated cell numbers = 347); Flot-1-CA-GFP-expressing group (FBS-untreated cell numbers = 348, FBS-treated cell numbers = 304); and Flot-1-CA/KR-GFP-expressing group (FBS-untreated cell numbers = 423, FBS-treated cell numbers = 447).

### Gene expression profiles and data analysis

Data used for the measurement of expression of EMT-related genes, as well as *FLOT1* and *UBE2I*, in human benign prostate and primary and metastatic prostate cancer tissues, originally from Varambally et al. [30], were downloaded from the GEO database (accession number: GSE3325). Total 19 individual benign prostate (*n* = 6), primary (*n* = 7), and metastatic (*n* = 6) prostate cancer samples were analyzed by the scatter plot using GraphPad Prism 5.0 version (GraphPad Software) for *FLOT1* and *UBE2I* expression. Total 6 pooled samples from benign (*n* = 2; NX1, NX2), primary (*n* = 2; PX1, PX2), or metastatic (*n* = 2; WX1, WX2) prostate cancer tissues were analyzed by the heat map of log_2_ gene expression for the EMT-related genes induced by Snail using Morpheus software (https://software.broadinstitute.org/morpheus/).

### Statistical analysis

Data are expressed as mean ± s.d. and analyzed in GraphPad Prism 5.0 version (GraphPad Software). Statistical analysis of more than two groups was performed by one-way (see Fig. 6d) and two-way ANOVA (see Figs. 1c, 2c, 3a, c, g, h, 4a, c–e, 5a–e, and 6a) followed by Bonferroni post-hoc test. Analysis comparing two groups was carried out using an unpaired Student’s *t* test (see Figs. 2a, b, d, e, 3d–f, 4b, and 6b). *P* values < 0.05 were considered statistically significant (*P < 0.05, **P < 0.01, ***P < 0.001).

## Supplementary information

Supplementary data

Supplementary Figure S1

Supplementary Figure S2

Supplementary Figure S3

Supplementary Figure S4

Supplementary Table 1

Supplementary Table 2
