# Supplementary material for: Sumoylation of Flotillin-1 promotes EMT in metastatic prostate cancer by suppressing Snail degradation
Source: Oncogene. 2019 Jan 10;38(17):3248–60. doi: 10.1038/s41388-018-0641-1 (PMC6756018; doi:10.1038/s41388-018-0641-1)
Supplement: Supplementary file 1 — Supplementary data [file 41388_2018_641_MOESM1_ESM.doc]

**Supplementary data**

**Materials and methods**

**Cell culture**

Human prostate adenocarcinoma DU145 cells, derived from brain metastasis were purchased from the Korean Cell Line Bank. Cells were cultured in RPMI-1640 medium (Thermo Fisher Scientific) containing 10 mM D-glucose supplemented with 10% FBS (ATCC) and 1% penicillin/streptomycin in a 5% CO_2_ incubator at 37 ˚C. For mitogen stimulation, cells were serum-deprived for 24 h and treated with or without 10% FBS for 6 h. Cells were routinely tested for negative mycoplasma contamination using Mycoplasma Detection Kit (Lonza).

**MTT assay**

The methylthiazolyldiphenyl-tetrazolium bromide (MTT, Sigma-Aldrich) assay was performed as described previously (3). Cells were treated with 0.5 mg/ml MTT solution at 37 °C for 4 h. The cells were then incubated with 100 μl of DMSO (BioShop) at 37°C for 30 min to solubilize the final product of MTT metabolism, the formazan precipitate. The optical density of each well was measured using a microplate reader set to 540 nm (Model 550, Bio-Rad Laboratories).

**Figure legends**

**Fig. S1. Mitogen-response Flot-1 sumoylation with up-regulated UBC9 correlates with up-regulation of Snail in DU145 and PC3 prostate cancer cells with high metastatic potential.**

**a** Up-regulation of UBC9 and Snail in prostate cancer cells with high metastatic potential. Immunoblot analysis of De-WCL from LNCaP, DU145, and PC3 cells stimulated with (FBS) or without (Cont) 10% FBS for 6 h. The densitometry ratios of Snail, UBC9, Flot-1, and SUMO-2/3 to actin are illustrated (n.s., not significant; mean±s.d., *n*=3, *^*^P*<0.05, *^**^P*<0.01, two-way ANOVA).

**b** Mitogen-induced sumoylation of Flot-1 in DU145 cells. Immunoblot analysis of De-IP performed with anti-Flot-1 antibody from DU145 cells stimulated with or without 10% FBS for 6 h. The densitometry ratios of sumoylated Flot-1 to unmodified Flot-1 are illustrated (mean±s.d., *n*=3, *^*^P*<0.05, Student’s *t* test).

**Fig. S2. Sumoylation of Flot-1 at Lys-195 is important for nuclear Snail stability.**

Immunoblot analysis of non-nuclear (nN) and nuclear (N) fractions from GFP-vector (Vec)-, WT Flot-1-GFP-, Flot-1-K51R-GFP-, Flot-1-K195R-GFP-, or Flot-1-KR-GFP-expressing PC3 cells stimulated with or without 10% FBS for 6 h. The amounts of Snail, ectopic WT Flot-1-GFP, Flot-1-K51R-GFP, Flot-1-K195R-GFP, and Flot-1-KR-GFP present in nuclear fraction are presented with 100% representing the total (nN + N) fractions (mean±s.d., *n*=3, *^**^P*<0.01, *^***^P*<0.001, two-way ANOVA).

**Fig. S3. Sumoylation of Flot-1 at Lys-195 is crucial for interaction with PTOV1.** Immunoblot analysis of IP performed with anti-GFP antibody and WCL from GFP-vector (Vec)-, WT Flot-1-GFP-, Flot-1-K51R-GFP-, Flot-1-K195R-GFP-, or Flot-1-KR-GFP-expressing PC3 cells stimulated with or without 10% FBS for 6 h. The densitometry ratios of Flot-1 interaction with PTOV1 are illustrated (n.s., not significant; mean±s.d., *n*=3, two-way ANOVA).

**Fig. S4. Sumoylation of Flot-1 regulates IGF-1-induced migration, but not proliferation, of metastatic PC3** **prostate cancer cells.**

**a** Palmitoylation, but not sumoylation, of Flot-1 is involved in IGF-1- and mitogen-induced proliferation of metastatic PC3 prostate cancer cells. MTT assay of GFP-vector (Vec)-, WT Flot-1-GFP-, Flot-1-KR-GFP-, or Flot-1-CA-GFP-expressing PC3 cells stimulated with or without 10 nM IGF-1 or 10% FBS for 24 h. Data are represented as mean±s.d (n.s., not significant; *n*=3, *^***^P*<0.001, two-way ANOVA).

**b** Sumoylation of Flot-1 promotes IGF-1-stimulated migration of metastatic PC3 prostate cancer cells. Migration assay of GFP-vector (Vec)-, WT Flot-1-GFP-, and Flot-1-KR-GFP-expressing PC3 cells stimulated with or without 10 nM IGF-1 for 24 h. The area of migrated cells in response to serum stimulation based on the obtained images was presented with 100% representing the vector control (mean±s.d., *n*=9, *^*^P*<0.05, *^**^P*<0.01, two-way ANOVA).
