# Supplementary figures and images for: Sumoylation of Flotillin-1 promotes EMT in metastatic prostate cancer by suppressing Snail degradation
Source: Oncogene. 2019 Jan 10;38(17):3248–60. doi: 10.1038/s41388-018-0641-1 (PMC6756018; doi:10.1038/s41388-018-0641-1)

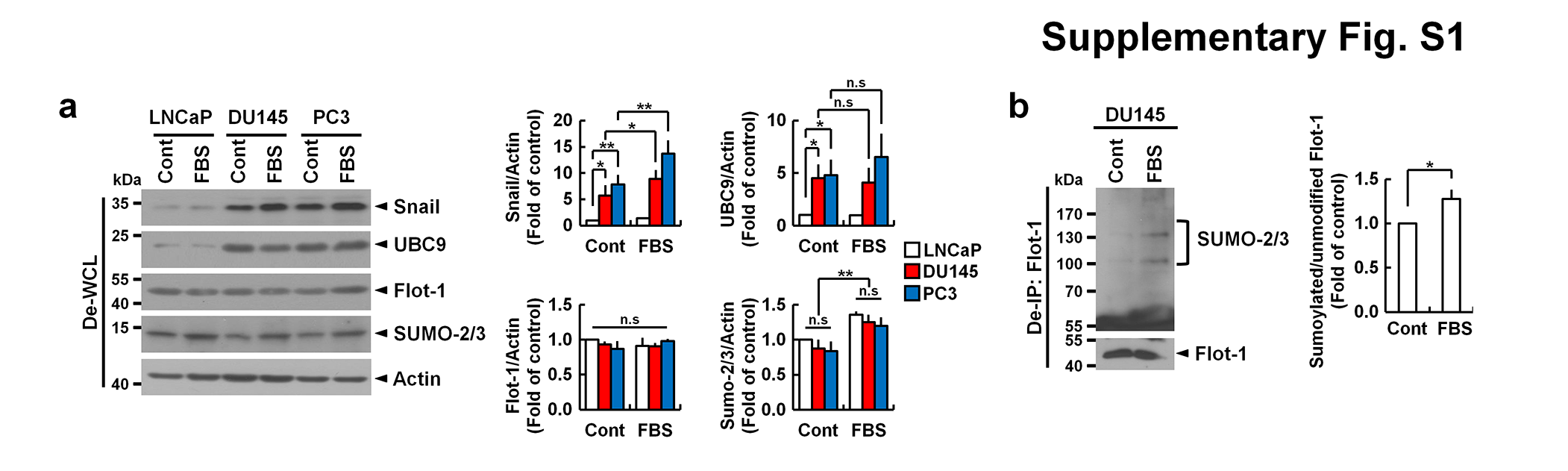

Supplement: Supplementary file 2 — Supplementary Figure S1 [file 41388_2018_641_MOESM2_ESM.tif]

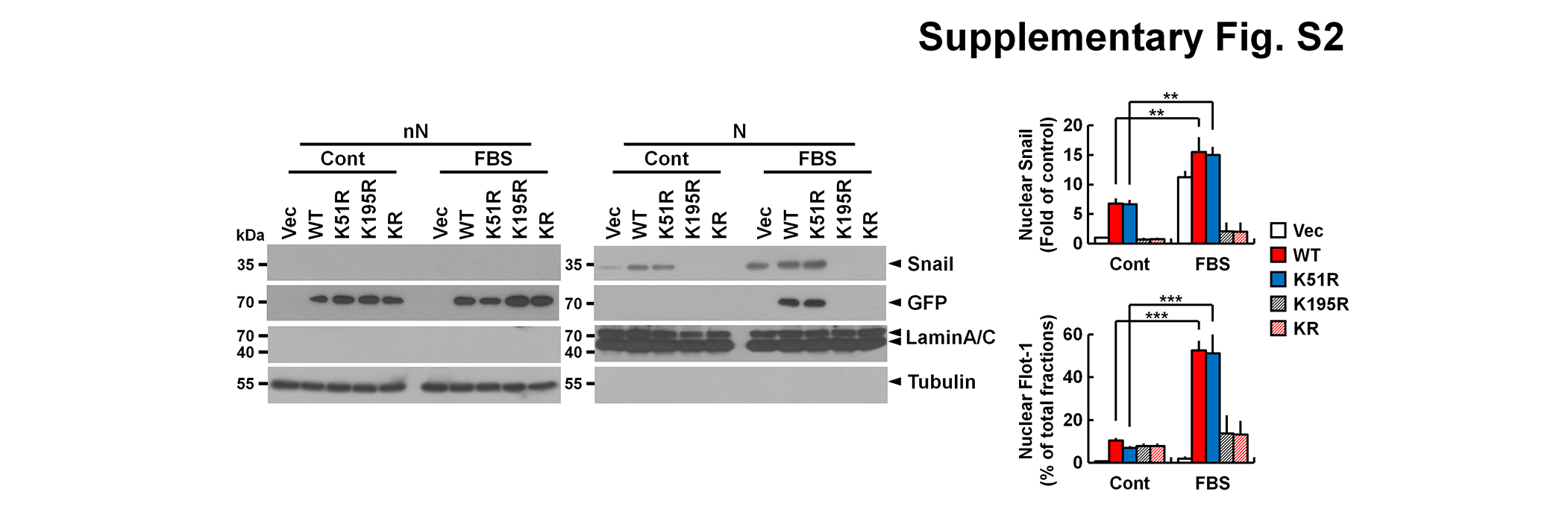

Supplement: Supplementary file 3 — Supplementary Figure S2 [file 41388_2018_641_MOESM3_ESM.tif]

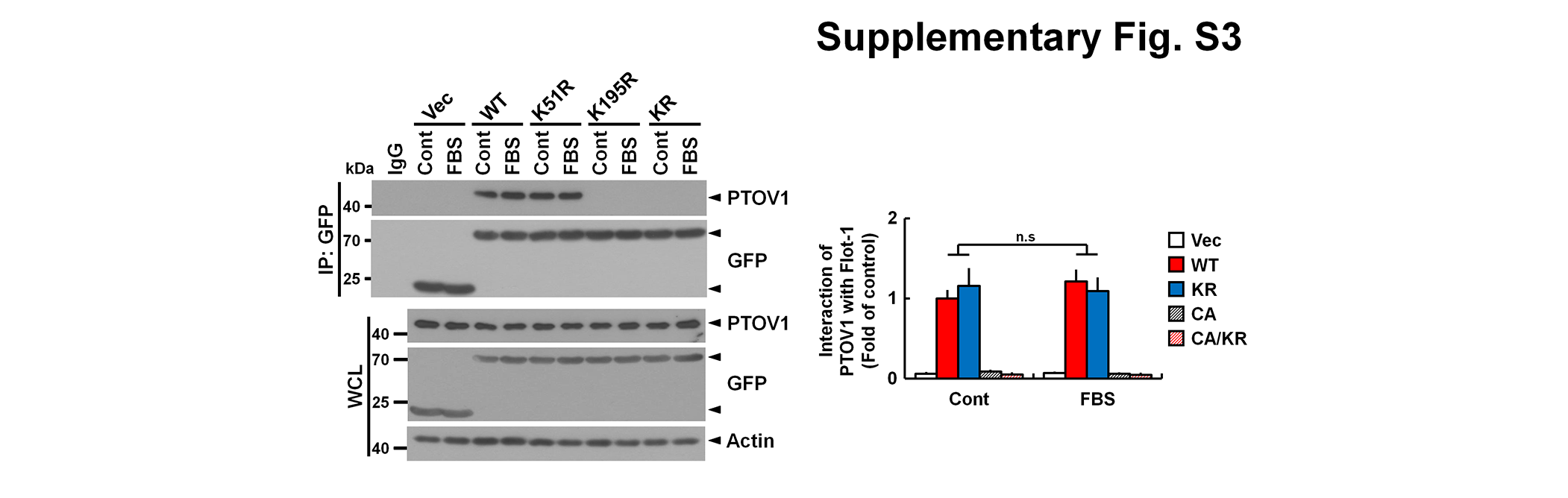

Supplement: Supplementary file 4 — Supplementary Figure S3 [file 41388_2018_641_MOESM4_ESM.tif]

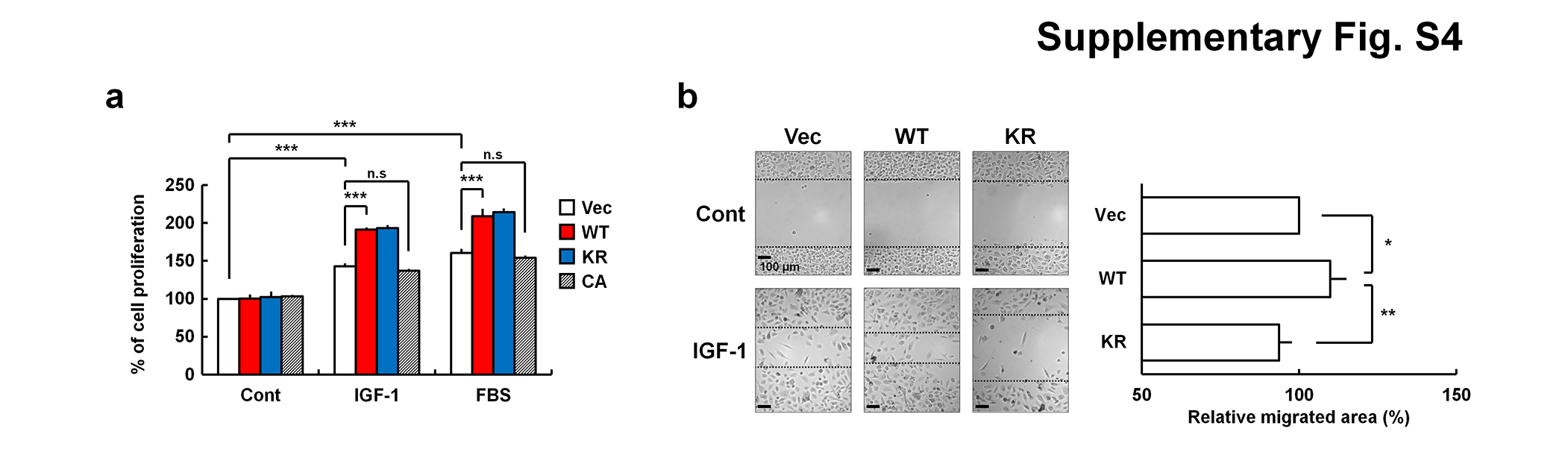

Supplement: Supplementary file 5 — Supplementary Figure S4 [file 41388_2018_641_MOESM5_ESM.tif]
